# Physical and autonomic functionality in women with breast cancer pre and post chemotherapy: a case control study

**DOI:** 10.1186/s13102-023-00797-y

**Published:** 2024-01-02

**Authors:** Bruna Lorena Soares Cavalcante Sousa, Thiago dos Santos Rosa, Valmir Oliveira Silvino, Esmeralda Maria Lustosa Barros, Hugo de Luca Corrêa, Danilo Marcelo Leite do Prado, Acácio Salvador Veras-Silva, Mariana Duarte de Souza, Carlos Eduardo Batista de Lima, Luciano Fonseca Lemos de Oliveira, Marcos Antonio Pereira dos Santos

**Affiliations:** 1 Nucleus of Study in Physiology Applied to Performance and Health (NEFADS), Federal University of Piaui, Teresina, PI Brazil; 2 Rehabilitation Sciences Program, Federal University of Minas Gerais, Belo Horizonte, MG Brazil; 3 Department of Physical Education, Catholic University of Brasilia, Brasilia, DF Brazil; 4 Rede Nordeste de Biotecnologia (RENORBIO), Federal University of Piaui, Teresina, PI Brazil; 5 Department of Biophysics and Physiology, Federal University of Piaui, Teresina, PI Brazil; 6 University of São Paulo, School of Medicine, São Paulo, SP Brazil; 7 Clinical Research Center of the University Hospital, Federal University of Piaui, Empresa Brasileira de Serviços Hospitalares (EBSERH), Teresina, PI Brazil; 8 Department of Applied Physical Therapy, Federal University of Triangulo Mineiro, Uberaba, MG Brazil; 9 University Federal of Minas Gerais (UFMG), 641 Ministro Pedro Borges Street, Tabuleta, Teresina, PI 64019650 Brazil

**Keywords:** Cardiotoxicity, Physical functional performance, Exercise test, Breast neoplasms

## Abstract

**Background:**

Breast cancer (BC) is one of the most incident types of cancer among women in the world. Although chemotherapy is an effective way to treat several types of cancer, it may also cause serious complications, including cardiotoxicity. This study aimed to identify the impact of chemotherapy on functional capacity, muscle strength and autonomic function.

**Methods:**

Ten breast cancer patients in therapeutic follow-up (TG) and ten women without comorbidities (CG) participated in the study (46±8.87 years old). Both groups were evaluated at two time points, before and 20 weeks after the start of chemotherapy. Functional capacity and muscle strength were assessed by 6-minute walk test (6MWT) and handgrip test, respectively. Autonomic function was assessed by heart rate variability analysis.

**Results:**

TG presented greater reductions in the handgrip test for the non-dominant hand (TG ↓15.2%; CG: ↑1.1%, *p*<0.05) compared to GC. However, no significant differences were found regarding VO_2_max (*p*>0.05) and 6MWT total distance (*p*>0.05). Regarding the heart rate variability variables before and after follow-up period, rMSSD (CG= 39.15±37.66; TG= 14.89±8.28, *p*= 0.01) and SDNN (CG= 55.77±40.03; TG= 26.30±10.37, *p*= 0.02) showed effect in the group and time interaction, whereas the LF/HF ratio presented significant difference only in the time analysis (CG= 2.24±2.30; TG= 2.84±1.82, *p*= 0.04).

**Conclusion:**

Chemotherapy used in the treatment of breast cancer patients resulted in decreased muscle strength and autonomic imbalance. The data suggests that chemotherapy may carry the risk of developing cardiovascular disease.

**Trial registration:**

Registration not required.

**Supplementary Information:**

The online version contains supplementary material available at 10.1186/s13102-023-00797-y.

## Background

Cancer incidence and mortality rates are rapidly increasing worldwide [1]. In 2020, 2.3 million new cases of breast cancer (BC) were registered [1–3]. As it is the second leading cause of cancer-related death in women, it is considered a serious public health problem [4]. In Brazil, BC is among the most frequent types of cancer in all Brazilian regions, and it is estimated that there will be 73,610 new cases in the period 2023/2025 with an incidence rate of 66.54 cases per 100,000 Brazilian women [3].

Regardless of the molecular subtype, whether luminal A, B, human epidermal growth factor receptor (HER) type 2 or triple negative, with the development and success of BC treatment in recent decades, it is known that its survival rate after five years is around 85% in developed countries and 60% in developing countries [5]. However, the cardiotoxic effects of antineoplastic have increased morbidity and mortality due to cardiovascular diseases, which justifies the growing interest of researchers in identifying the correct management of the treatment [3, 6]. Whether adjuvant or neoadjuvant, chemotherapeutic strategies to treat BC pose a risk to the health of the patient [7–11]. Antimicrotubule agents, anthracyclines and HER-directed therapies are well known for being related to ventricular dysfunction and heart failure [10]. Therefore, early cardiotoxicity identification may improve cardiac and oncology management [6, 12, 13].

The echocardiography is a widely used technique to assess cardiotoxicity that is defined as a ≥ 10% reduction in left ventricular ejection fraction (LVEF) to a value below the lower limit of normality (LVEF < 50%) [10]. However, sympathetic overdrive or increase in LV preload can compensate the LVEF in the initial stages of cardiac aggression [14]. Therefore, the assessment of the autonomic function may be an alternative evaluation as it plays a fundamental role in the regulation of heart rate, myocardial function, and myocardial blood flow [13, 14].

Thus, the therapeutic phase of BC requires special attention regarding the identification of possible cardiovascular risk factors associated with exercise capacity and autonomic function. This strategy may prevent further health problems and minimize care costs [7]. It is hypothesized that BC treatment may contribute to lower tolerance to effort, decreased peripheral muscle strength and autonomic dysfunction, which may lead to cardiac complications. Thus, this study aimed to evaluate the effects of chemotherapy on the functional capacity and autonomic function in women with breast cancer.

## Methods

### Study design

This is a prospective, case-control study. The group that underwent therapeutic follow-up with chemotherapy (TG) consisted of women with a recent diagnosis of BC with the prospect of starting treatment with neoadjuvant and adjuvant chemotherapy [weekly Pactaxel, associated with the Doxorubicin + Cisplatin (AC) regimen for 15 days or Trastuzumab for 21 days]. as described in the Brazilian Cardio-oncology Guideline – 2020 [10]. A control group (CG) of age-matched healthy women was also investigated.

### Participants

The sample size was calculated based on the results of the study by Gonzaga et al., 2018 [15]. Using the mean and standard deviation of the SDNN variable from both groups resulted in an effect size of 1.54. Adopting an α error of 0.05 and a statistical power (β error) of 0.80, a minimum sample size of 6 volunteers per group was determined.

Participants were recruited between March and August 2019, and the follow-up period extended until February 2020. The following eligibility criteria for the TG were considered: recent diagnosis of BC, expected start of neoadjuvant or adjuvant chemotherapy to the therapeutic protocol, aged over 18 years, clinical stability and medical authorization to perform the functional tests. Volunteers were excluded if: unable to perform the scheduled chemotherapy protocol; previous diagnosis of chronic obstructive pulmonary disease; heart failure; infectious diseases; unable to understand or fail to complete the assessments; or unable to contact for reassessment.

The control group, consisting of women without breast cancer, was recruited through social media advertising. We included healthy women without comorbidities, matching the age range and anthropometric measurements of the breast cancer group. As this is an observational study, participants did not engage in an exercise intervention. This case-control study was approved by the ethics committee of the Federal University of Piaui, under protocol 3.131.097. All participants signed the informed consent form and all procedures were carried out in accordance with the guidelines proposed by resolution 466/12 of the National Health Council.

### Measures

The data collection for this study occurred at the Biophysics and Physiology Laboratory at UFPI (Federal University of Piauí), equipped with the necessary infrastructure for conducting the required assessments. The collection was conducted from April 2019 to February 2020, strategically planned to cover a comprehensive and relevant timeline for breast cancer treatment.

On average, each participant underwent assessments for approximately 150 days, corresponding to the standard 5-month duration of breast cancer chemotherapy treatment. The initial assessment took place before the commencement of chemotherapy, and the subsequent reassessment occurred after the completion of the treatment.

The sociodemographic variables were acquired by individual questionnaire (Supplementary file 1 provides the variables evaluated in this study) while clinical variables (clinical staging, histological type, comorbidities, type of chemotherapy and therapeutic combination) were withdrawn from the patient’s record.

Four researchers, previously trained for data collection, served as outcome assessors. They were blinded to minimize subjective interpretations and possible bias. One researcher assessed autonomic function, two evaluated the functional capacity and one evaluated the muscle strength. All measurements were performed by the same evaluators before and after the follow-up period. Autonomic function was assessed first on the day of collection, followed by muscle strength and, finally, functional capacity. All measurements were conducted in the morning and in the same environment, free of noise and with a controlled temperature between 22ºC and 24ºC.

### Functional capacity

The functional capacity was investigated by a 6-minute walk test (6MWT) performed in a 30 meters corridor, according to American Thoracic Society guideline [16]*.* During the evaluation, each participant performed two tests to eliminate the learning effect and ensure the reproducibility of the procedure, with a minimum rest interval of 15 minutes between tests. To record the data, the results of the second test were considered. Considering the female gender = 0 and the delta of heart rate (∆HR), we estimated the expected walking distance of the test (6MWD) for each participant with the following equation proposed by Britto et al., 2013 [17]:

$$6{\text{MWD}}=356.658-\left(2.303\times {{\text{age}}}_{{\text{years}}}\right)+\left(36.648\times {\text{sex}}\right)+\left(1.704\times {{\text{height}}}_{{\text{cm}}}\right)+\left(1.365\times \Delta {\text{HR}}\right)$$

For the peak oxygen uptake (VO_2_peak) estimation, we used the following equation proposed by American College of Sports Medicine Guidelines for stress testing and exercise prescription [18]:

$${\text{VO}}_2\text{peak}\;est=\left(\left(0.02\times{6\text{MWD}}_\text{m}\right)-\left(0.191\times{\text{age}}_\text{years}\right)-\left(0.07\times{\mathrm{body\;mass}}_\text{kg}\right)+\left(0.09\times{\text{height}}_\text{cm}\right)+\left(0.26\times\text{RPP}\times10^{-3}\right)+2.45\right)$$

### Muscle strength

The muscle strength was assessed by a handgrip test using a manual dynamometer. The measurement was performed by using the Crown ® 100 dynamometer to the nearest 0.01 kg. The patients performed the test in a sitting position, with the elbow flexed at a 90º angle, with forearm and wrist in neutral position. Participants were instructed to perform three maximal isometric contractions, with 30 seconds of interval between measurements, with the time being estimated by a stopwatch to standardize the rest for each participant. Three measurements were obtained from each hand and the average was grouped as dominant and non-dominant hand [19]. The values obtained were classified according to age. Values below the fifth percentile (P5) were classified as muscle strength depletion [20].

### Autonomic function

The autonomic function was assessed through the monitoring of heart rate variability (HRV) using the Polar H10 heart monitor (Polar OY, Finland), previously validated at rest and during exercise [21]. The device was placed on the thoracic waist using an elastic chest strap below the pectoral muscles of each participant. They were instructed to wear comfortable clothing, not speak or move during the analysis, and remain in a lying position for 15 minutes while recording the RR intervals. The heart rate monitor was connected to the Android smartphone app HRV Expert by CardioMood. The data recorded by the app were exported as a text file and analyzed by Kubios HRV Standard software (version 3.2.0; Biosignal Analysis and Medical Image Group, Department of Physics, University of Kuopio, Kuopio, Finland) [22].

Data were analyzed in the time and frequency domains, as measures of electrical activity from parasympathetic and sympathetic impulses of autonomic balance. In the time domain, we analyzed the root mean square of successive differences between RR intervals (rMSSD), which was selected to reflect changes in vagal modulation and was preferred due to its greater reliability in demonstrating parasympathetic activity when compared to other indices of power spectral density; and the standard deviation of all normal RR intervals (SDNN) which represents the contribution of the sympathetic and parasympathetic components of the autonomic nervous system. Regarding the frequency domain, the low frequency/high frequency ratio (LF/HF) was chosen to analyze the autonomic balance, regarding the LF as 0.04 to 0.15 Hz and HF as 0.15 to 0.4 Hz [23].

### Statistical analysis

There were no missing data in our statistical analysis. All data collected were duly recorded and included in the analysis. Data were presented as mean and standard deviation. Normality and homogeneity of the data were verified using the Shapiro-Wilk and Levene tests, respectively. Independent t test was used to compare means and mixed ANOVA test was used to compare means and mixed ANOVA test was used to analyze variables between groups with and without breast cancer, as well as to analyze changes over time measures with Least significant difference (LSD) post hoc test. Statistical significance level was established at p<0.05. The analysis was performed using the Statistical Package for the Social Sciences (SPSS ®, version 21.0) for Windows.

## Results

**Fig. 1:**
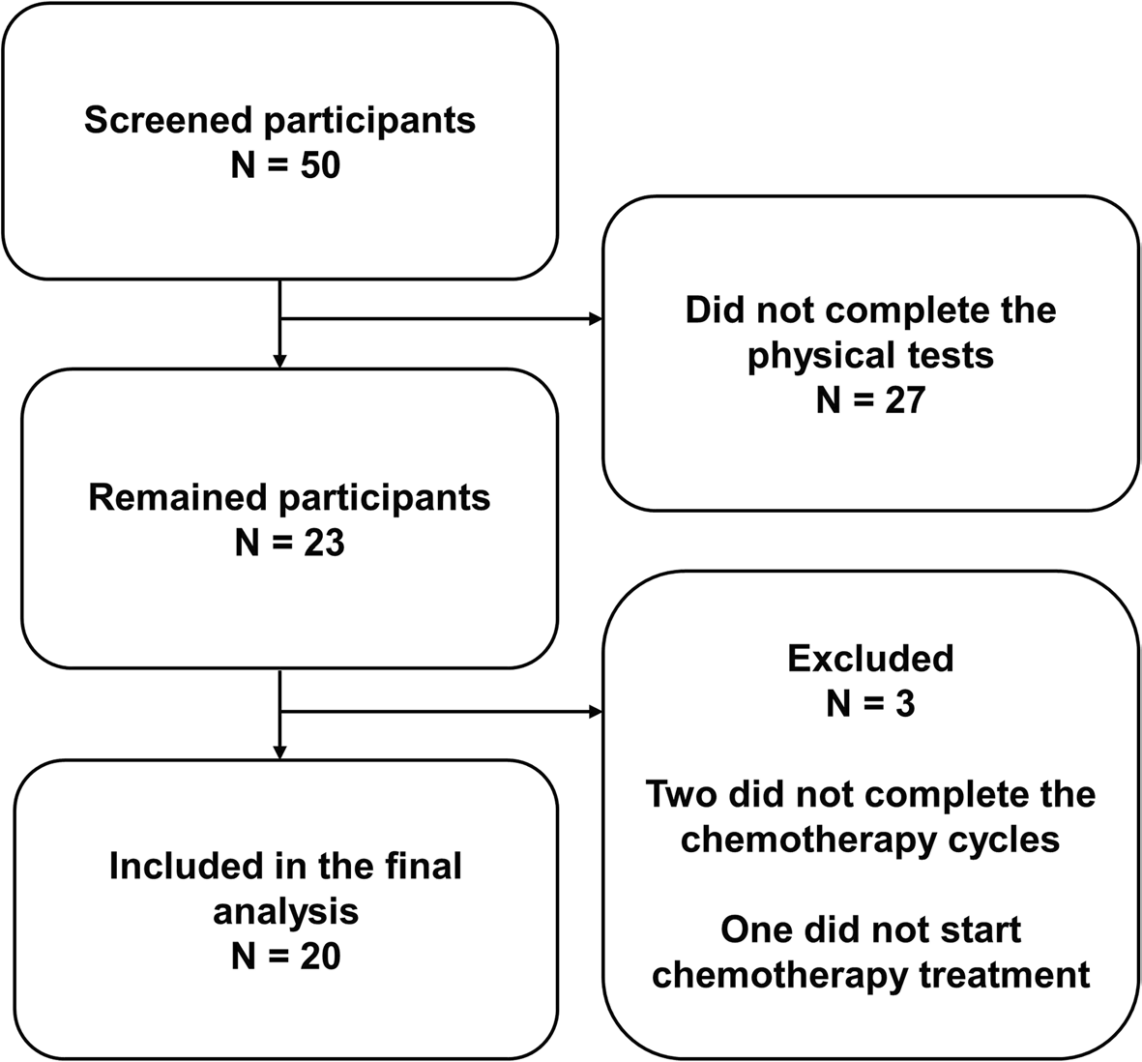
Flow chart of the participants

Figure 1 shows the progression of participants throughout the study. Firstly, fifty participants were screened. However, twenty-seven were excluded as they were not able or decided not to perform the physical tests. In total, twenty-three participants started the program, and of those, three were excluded as they did not start or complete the chemotherapy cycles. The data of the 20 completers were included in the final analysis. 10 BC participants comprised the TG and 10 women at the same age range without BC comprised the CG.

Regarding the clinical variables, the participants in the CG did not have associated comorbidities. The invasive lobular was the predominant histological tumor type, representing 60% of the sample while 40% of the volunteers of the TG were diagnosed with infiltrating ductal carcinoma. The most frequent molecular classifications were Luminal A (50%), triple negative (30%) and Luminal B (20%). Regarding the clinical stage of BC, 90% of the participants were in Stage I and 10% in Stage II. Sixty percent of the participants started treatment with neoadjuvant chemotherapy and 40% underwent surgery before chemotherapy. Eighty percent underwent the chemotherapy regimen with Doxorubicin + Cisplatin (AC) (every 15 days) + Paclitaxel (weekly) while 20% used Paclitaxel (weekly) + Trastuzumab (every 21 days) as described in Table 1.

**Table 1:** Clinical profile of the therapy group

Clinical variables	TG
**Histological type**	
Infiltrating ductal carcinoma	4 (40)
Invasive lobular	6 (60)
**Immunohistochemistry**	
ER+	5 (50)
PR+	5 (50)
HER2+	1 (10)
Ki67 <20%	7 (70)
Ki67≥20%	2 (20)
**Molecular subtype**	
Luminal A	5 (50)
Luminal B/HER2	2 (20)
Triple negative	3 (30)
**Staging**	
I	9 (90)
II	1 (10)
**Chemotherapy**	
Neoadjuvant	6 (60)
Adjuvant	4 (40)
**Protocol**	
AC (15 days) + Taxol (Weekly)	8 (80)
Taxol (Weekly) + Herceptin (every 21 days)	2 (20)

Date presented as mean ± standard deviation or absolute frequency (relative frequency). Legends: *TG* Therapy Group, *ER* Estrogen Receptor, *PR* Progesterone Receptor, *HER* Human epidermal growth factor receptor type 2; *Ki67* Antigen-marker of cell proliferation, *AC* Doxorubicin + Cisplatin, *Taxol* Paclitaxel, *Herceptin* Trastuzumab

The TG presented similar age (TG: 47.9±8.4; CG: 44.1±9.34 years, *p*= 0.35), body mass (TG: 68.6±11.1; CG: 66.2±9.2 kg, *p*= 0.61) and body mass index (TG: 26.59±4.34; CG: 26.43±2.55 kg/m^2^, *p*= 0.92) when compared to CG. Table 2 show the results obtained from the cardio-respiratory, muscle strength and autonomic parameters assessed pre-follow-up period. When comparing means of the total distance (TG:473.55±79.81m; CG: 437.00±39.26m, *p*= 0.21), dominant muscle strength (TG: 23.37±2.66; CG: 25.05±4.41, *p*=0.32) and ratio LF/HF (TG: 1.22±0.47; CG: 2.02±1.16, *p*=0.07) the groups presented equal values.

**Table 2:** Main anthropometrics, cardio-respiratory, muscle strength and autonomic parameters assessed pre-follow-up period.

**Anthropometrics**	TG (*n*= 10)	CG (*n*= 10)	Mean difference (95% CI)	P
Age (years)	47.90±8.41	44.10±9.34	3.80 (-4,55;12,15)	0.35
Body mass (Kg)	68.62±11.13	66.24±9.23	2.38 (-7.23;11.99)	0.61
BMI (Kg/m^2^)	26.59±4.34	26.43±2.55	0.16 (-3.19;3.51)	0.92
**Cardio-respiratory parameters**				
Total distance (m)	473.55±79.81	437.00±39.26	36.55 (-22.54;95.64)	0.21
% of estimated distance (%)	83.72±15.02	76.51±6.87	7.21 (-3.76;18.19)	0.18
VO2 (ml·Kg·min^-1^)	16.65±1.65	15.97±1.84	0.69 (-0.95;2.33)	0.39
**Muscle strength**				
Dominant (Kg)	23.37±2.66	25.05±4.41	-1.68 (-5.10;1.74)	0.32
Non-dominant (Kg)	20.17±3.28	22.18±3.70	-2.01 (-5.30;1.28)	0.21
**Autonomic function**				
rMSSD	44.43±25.27	28.77±13.64	15.66 (-3.42;34.74)	0.10
SDNN	72.14±61.37	41.72±13.64	30.42 (-13.94;74.79)	0.16
LF/HF	1.22±0.47	2.02±1.16	-0.80 (-1.66;0.07)	0.07

Values are expressed as mean ± standard deviation

*Abbreviations*: *TG* Therapy Group, *CG* Control Group, *N* Number, *BMI* Body Mass Index, *M* Meters, *VO2max* Maximal oxygen uptake, *Ml* Milliliters, *Kg* Kilogram, *Min* Minute, *rMSSD* Root mean square of successive differences between RR intervals, *SDNN* Standard deviation of all RR intervals, *LF* Low Frequency, *HF* High Frequency

Table 3 presents the descriptive results of the assessment before and after follow-up period. A time effect was verified on the cardiorespiratory variables (Total distance (m); % of estimated distance (%); VO_2_ (ml·Kg·min^-1^) *p*<0.05. Additionally, there was a significant decrease in the strength of the dominant hand (*p*<0.05) after the breast cancer treatment period. In contrast, the between-subject effect was identified only for the non-dominant hand (*p*<0.05). The group and time interaction influenced maximal isometric strength for the dominant hand and non-dominant (*p*<0.05). Autonomic function variables, rMSSD and SDNN, showed a significant difference in the group and time interaction (*p* <0.05), except the LF/HF ratio (*p* >0.05) which had an effect only on the time factor.

**Table 3:** Cardio-respiratory, muscle strength and autonomic parameters assessed before and after follow-up period

	TG (*n*= 10)		CG (*n*= 10)				*P* values		
Variables	PRE	POST	Mean difference (95% CI)	PRE	POST	Mean difference (95% CI)	G	T	G x T
**Cardio-respiratory parameters**									
Total distance (m)	473.55±79.81	421.20±75.06^†^	52.35 (10.33;94.37)	437.00±39.26	398.5±94.87	38.5 (-3.52; 80.52)	0.34	**<0.01**	0.63
% of estimated distance (%)	83.72±15.02	75.06±12.99^†^	8.67 (1.44;15.89)	76.51±6.87	69.63±16.58	6.88 (-0.34;14.11)	0.26	**<0.01**	0.72
VO2 (ml·Kg·min-1)	16.65±1.65	15.60±2.24^†^	1.06 (0.54;2.06)	15.97±1.84	15.58±2.31	0.39 (-0.61;1.39)	0.68	**<0.05**	0.34
**Muscle strength**									
Dominant	23.37±2.66	19.39±4.66^†^	3.98 (2.38;5.58)	25.05±4.41	23.35±4.66^†^	1.70 (0.09;3.30)	0.10	**0,00**	**<0.05**
Non-dominant hand	20.17±3.28	17.10±3.51^†^	3.07 (0.91;5.23)	22.18±3.70	22.43±5.25	-0.25 (-2.41;1.91)	**0.04**	0.07	**0.03**
**Autonomic function**									
rMSSD	44.43±25.27	14.89±8.28^†^	29.54 (7.63;51.46)	28.77±13.64	39.15±37.66	-10.37 (-32.29;11.54)	0.59	0.21	**0.01**
SDNN	72.14±61.37	26.30±10.37^†^	45.84 (11.01;80.67)	41.72±13.64	55.77±40.03	-14.04 (-48.87;20.78)	0.97	0.19	**0.02**
LH/HF	1.22±0.47	2.84±1.82^†^	-1.62 (-2.87;-0.37)	2.01±1.16	2.24±2.30	-0.23 (-1.48;1.02)	0.86	**0.04**	0.12

Values are expressed as mean ± standard deviation

*Abbreviations*: *TG* Therapy Group, *CG* Control Group, *N* Number, *M* Meters, *VO*_*2*_*max* Maximal oxygen uptake, *Ml* Milliliters; *Kg* Kilogram, *Min* Minute; *rMSSD* Root mean square of successive differences between RR intervals, *SDNN* Standard deviation of all RR intervals, *LF* Low Frequency, *HF* High Frequency

*P* value from the mixed ANOVA analysis of variance are listed as group effect (G), time effect (T) and groups x time effect(G x T); †: Post-hoc test identifies significance (*P* ≤0.05) in differences between PRE and POST

## Discussion

To our knowledge, this is the first case control study that evaluated the exposure of chemotherapy on autonomic function and functional capacity in breast cancer patient. Importantly, this study included breast cancer patients with an average age of 47.9 years old. This scenario is adverse because there is a worldwide increase in this neoplasm in women under 40 years old, with advanced age associated with a worse prognosis [24, 25]. The participants of the TG were characterized with small breast tumors, favorable biological characteristics, and stage I or II at diagnosis, which are associated with fewer adverse outcomes during treatment [26].

Peripheral muscle strength and autonomic function are associated as factors that can potentially influence the functional capacity of women with BC during chemotherapy [27–30]. Accordingly, Gupta et al., 2008 [31] stated that patients with depleted handgrip strength are conditioned to greater risks of adverse health outcomes. An isometric dynamometer, the device widely used to perform the handgrip test, is accessible, practical, and has high predictive power and, thus, can be included in the routine evaluation of BC patients. In the present study, there was a significant decrease in peripheral muscle strength for the non-dominant limb, with the risk of muscle depletion being classified in 20% of the post-chemotherapy sample. In another research involving cancer patients, it was observed that the decrease in peripheral strength was associated with increased mortality, with clinical worsening when related to malnutrition and advanced age [32].

We conducted the 6MWT to evaluate the functional cardiorespiratory capacity, which is widely used in patients with chronic respiratory disease and heart failure [17, 31]. Importantly, this test has been used in several other populations, including cancer patients, with valid and reliable data [33]. Cheville et al., (2008) [34] conducted a study with 163 patients with metastatic BC and observed that 92% of them had physical impairments considered remediable. This observation was justified due to the combination of more than one casual factor, including stage of the disease or side effect of the surgical, chemotherapy and/or radiotherapy treatment. Likewise, they observed a shorter total distance and functional capacity after the chemotherapy treatment.

Regarding the equation by Britto et al., 2013 [17] the estimated distance was greater than the total distance covered after the 6MWT, not only before but also after chemotherapy, which may predict a decrease in functionality since diagnosis [28]. Therefore, considering the observational nature of this study, it was expected that there would be a decrease in oxygen consumption in the second assessment, as presented in the study by Wiestad et al, 2020 [35]. In a systematic review [36], the authors stated that the 6MWT total distance and VO_2_max can be improved with aerobic training, either continuous or intermittent, with a greater gain in the continuous modality. Additionally, individualized resistance training may improve these parameters when associated with aerobic training, based on the guidelines of the American College of Sports Medicine (ACSM) [18, 37, 38].

In this perspective, functional capacity is considered of paramount importance to ensure greater adherence and response to treatment, as well as to improve morbidity and mortality indicators [27]. It is explained that maintaining muscle mass and improving oxygen transport may favor a decrease in sympathetic tone and an increase in vagal tone, consequently decreasing heart rate (HR) at rest and increasing HRV and baroreflex sensitivity [14, 37–39]. This effect may be opposite in the absence of exercise practice [14]. Therefore, it is necessary to understand the possible functional impairments caused by chemotherapy during treatment. It should be considered the possibility of periodically monitoring the functional capacity and adapting treatment based on specific risk profiles. Additionally, the practice of physical activity should be encouraged, since it contributes to increase autonomy and independence, standards that are expected for adequate functionality [40–42].

Regarding the chemotherapy protocol used in the treatment of TG, it is known that anthracyclines are key components for the BC treatment, but they are limited in terms of cumulative and dose-dependent cardiotoxicity. The incidence of asymptomatic heart failure induced by doxorubicin is 2.2%, but it can increase to 27% when associated with trastuzumab. Of these, 16% had heart failure class III and IV based on the New York Heart Association functional classification [42]. It should be noted that both therapeutic protocols described in this study are worrying. The combination of doxorubicin and cyclophosphamide every 15 days associated with cycles with weekly paclitaxel, as well as weekly paclitaxel associated with trastuzumab every 21 days, seem to negatively contribute to autonomic imbalance, leading cardiotoxicity that can have a critical and negative impact on functionality [28]. However, studies monitoring HRV in individuals with cancer are still scarce.

Since chemotherapy is cardiotoxic and may lead to functional impairments, the risks for the development of cardiovascular diseases should be taken into consideration. Treatment modification based on patient-specific risk profiles, intensive monitoring, detection of cardiovascular lesions by imaging and biomarkers circulation, and to pharmacological intervention with the prescription of cardioprotective drugs (i.e., beta-blockers or ACE inhibitors) are strategies widely used as preventive and protective measures in early detection and intervention of potential risks, although they are not always used in the clinical routine [42]. Another important and sensitive measure to be monitored concerns the autonomic variables, since it allows to verify whether the autonomic nervous system modulation is within normal range, which allows early detection of any alteration [15].

Similar to our study, Gonzaga et al., 2018 [15] also evaluated the autonomic modulation in patients with BC and compared them with a control group. However, their sample consisted of postmenopausal women who survived breast cancer using hormone therapy (aromatase inhibitors), which is also associated with increased risks of heart failure and cardiovascular. The results found were similar to those of our study, suggesting that HRV reduction is particularly evident in the LF oscillations band, with a statistically significant direct relationship between baroreflex impairment and HF band reduction. This is justified by the decline in myocardial function that is initially compensated for an increase in heart rate [14].

Fagundes et al., 2011 [43] found an association between HRV and fatigue in BC survivors within one year after treatment, which supported the hypothesis that reduced parasympathetic activity is associated with greater fatigue levels. In addition, De Couck and Gidron, 2013 [44] noted that the SDNN, rMSSD and HF band indexes were significantly lower in subjects with a more reserved prognosis, as found in women with breast cancer after chemotherapy, showing that individuals with better prognosis had a higher HRV, in parallel with the results found in the pre-chemotherapy phase.

Another relevant finding of our study refers to the handgrip strength test, which showed a consistent decline in functional status with autonomic imbalance after chemotherapy. This is probably due to the catabolic effects of chemotherapy, which may have directly affected the muscle fibers, with consequent reduction in muscle strength, thus impacting the global functionality [32, 45]. However, LF/HF ratio, peripheral muscle strength of the dominant hand and total 6MWT distance resulted in a significant difference only in the time analysis for both groups.

Therefore, health professionals should constantly assess functional and autonomic aspects of BC patients, as it can early detect possible adverse changes. Additionally, studies with HRV as a tool to screen autonomic modulation of BC patients undergoing chemotherapy are suggested. This can result in a more reliable overview of its applicability and effectiveness, since autonomic dysfunction is one of the main factors that may contribute to functional impairment and lower quality of life.

Despite the small sample size of our study, the number of participants is in accordance with the recommendation after the calculation of the statistical power. The selection of cardiac autonomic modulation as our primary outcome was based on its critical role in the study of cardiotoxicity. However, the decision to base the effect size calculation on the study by Gonzaga et al. 2018 [15] may introduce potential limitations. The aforementioned study is a cross-sectional study, and the authors did not evaluate baseline assessments as well as the potential role of important confounding factors (such as hypertension or chronic use of medications) that could affect autonomic modulation.

Our study is also limited to the fact that we included only participants diagnosed with stage I or II breast cancer, excluding those with metastases or associated comorbidities. This reflected in two different therapeutic strategies, as recommended by the Brazilian Guideline of Cardio-oncology 2020 [10]. It is noteworthy that there were no reports of serious adverse effects after initiation of chemotherapy by the participants in the TG. Although other comorbidities were not presented, which minimizes selection biases, other variables including adverse symptoms related to cancer treatment, sleep quality, and psychosocial factors may have interfered with the findings [14, 46]. Finally, the heterogeneity of tumor characteristics and antineoplastic treatments may have acted as confounding factors, also resulting in a limitation of the study.

It is worth mentioning that there are few investigations evaluating the oncological public in treatment with cardiotoxic chemotherapy, especially through the analysis of HRV, which is still recent in clinical practice. Therefore, our investigation reinforces the importance of comprehending the possible side effects of chemotherapy, highlighting the functional risks, the relevance of assessing physical and autonomic functionality, and the indispensable multidisciplinary follow-up during treatment. These strategies allow a more accurate diagnosis, aiming at more satisfactory health conditions in longer survival time and lower health costs.

## Conclusion

Both functional capacity and autonomic function may be compromised after chemotherapy due to the decrease in peripheral muscle strength and maximal oxygen consumption, revealing possible risks of muscle depletion and impairment of the cardiovascular system in breast cancer patients. Furthermore, chemotherapy may lead to sympathetic hyperactivity and parasympathetic hypoactivity. Although the findings of the autonomic dysfunction in the acute post-treatment phase, they may allow clarification on the therapeutic follow-up in a safe and satisfactory margin for women with breast cancer. Additionally, it may provide data for planning early intervention in the management of cardiotoxicity and prevention of cardiovascular diseases.

### Supplementary Information

**Additional file 1.** Socioeconomic questionnaire.

## Data Availability

The datasets analyzed in this manuscript are not publicly available but are available from the corresponding author on reasonable request.

## Abbreviations

BC: Breast cancer
TG: Therapy group
CG: Control Group
6MWT: 6-minute walk test
VO_2_max: Maximal oxygen uptake
rMSSD: Root mean square of successive differences between RR intervals
SDNN: Standard deviation of all normal RR intervals
LF: Low frequency
HF: High frequency
LF/HF ratio: Low frequency/high frequency ratio
HER: Human epidermal growth factor receptor
AC: Doxorubicin + Cisplatin
∆HR: Delta heart rate
6MWD: 6-minute walking distance
Cm: Centimeter
M: Meters
Kg: Kilogram
P5: Fifth percentile
HRV: Heart rate variability
RR: Time gap between 2 QRS complexes
Hz: Hertz
LSD: Least significant difference
SPSS: Statistical Package for the Social Sciences program
BMI: Body mass index
Kg: Kilogram
Cm: Centimeter
ACSM: American College of Sports Medicine
HR: Heart rate
ACE: Angiotensin-converting enzyme
