# Supplementary material for: Physical and autonomic functionality in women with breast cancer pre and post chemotherapy: a case control study
Source: BMC Sports Sci Med Rehabil. 2024 Jan 2;16:5. doi: 10.1186/s13102-023-00797-y (PMC10763182; doi:10.1186/s13102-023-00797-y)
Supplement: Supplementary file 1 — Additional file 1. Socioeconomic questionnaire. [file 13102_2023_797_MOESM1_ESM.docx]

**SOCIOECONOMIC QUESTIONNAIRE**

Date: ____/____/____

1. Current age: ____ years

2. Skin color:

(a) White

(b) Black

(c) Asian

(d) Brown

(e) Indigenous

(f) Other

(g) I prefer not to say

3. Marital status:

(a) Married in a civil or religious relationship

(b) Living in a stable marital union or in a domestic partnership

(c) Single

(d) Separated

(e) Divorced or legally separated

(f) Widowed

(g) I prefer not to say

5. Schooling:

(a) Never attended, does not know how to read or write

(b) Never attended, knows how to read and write

(c) 1st year of Elementary School

(d) 2nd year of Elementary School

(e) 3rd year of Elementary School

(f) 4th year of Elementary School

(g) 5th year of Elementary School

(h) 6th year of Elementary School

(i) 7th year of Elementary School

(j) 8th year of Elementary School

(k) 9th year of Elementary School

(l) 1st year of High School

(m) 2nd year of High School

(n) 3rd year of High School

(o) Incomplete technical courses at the secondary level

(p) Complete high school technical courses

(q) Incomplete higher education

(r) Completed higher education

(s) Incomplete post-graduation

(t) Completed post-graduation

(u) I prefer not to say

6. Do you currently have any activity, whether paid or unpaid?

(a) Yes, current employed

(b) Yes, but on sick leave

(c) Yes, but on maternity leave

(d) No, unemployed

(e) No, retired

(f) No, housewife

(g) No, pensioner

(h) No, only student

(i) Other

(j) I prefer not to say

7. In your main job, you are:

(a) Employee with statutory status or a formal employment contract

(b) Salaried employee without a formal contract

(c) Unpaid family servant

(d) Own or self-employed with an establishment

(e) Own or self-employed without an establishment

(f) Employer with up to 5 permanent employees

(g) Employer with more than 5 permanent employees

(h) Unpaid worker

(i) I prefer not to say

8. Total household income:

(a) _______ minimum wage(s) per month.

(b) I prefer not to say

9. Number of members residing in your current household:

(a) _____ people

(b) I prefer not to say
